# Developing and testing an instrument to measure the presence of conditions for successful implementation of quality improvement collaboratives

**DOI:** 10.1186/1472-6963-8-172

**Published:** 2008-08-11

**Authors:** Michel LA Dückers, Cordula Wagner, Peter P Groenewegen

**Affiliations:** 1 NIVEL – Netherlands Institute for Health Services Research, Utrecht, The Netherlands; 2 EMGO-Institute, Free University Medical Centre, Amsterdam, The Netherlands; 3 Department of Sociology, Department of Human Geography, Utrecht University, Utrecht, The Netherlands

## Abstract

**Background:**

In quality improvement collaboratives (QICs) teams of practitioners from different health care organizations are brought together to systematically improve an aspect of patient care. Teams take part in a series of meetings to learn about relevant best practices, quality methods and change ideas, and share experiences in making changes in their own local setting. The purpose of this study was to develop an instrument for measuring team organization, external change agent support and support from the team's home institution in a Dutch national improvement and dissemination programme for hospitals based on several QICs.

**Methods:**

The exploratory methodological design included two phases: a) content development and assessment, resulting in an instrument with 15 items, and b) field testing (N = 165). Internal consistency reliability was tested via Cronbach's alpha coefficient. Principal component analyses were used to identify underlying constructs. Tests of scaling assumptions according to the multi trait/multi-item matrix, were used to confirm the component structure.

**Results:**

Three components were revealed, explaining 65% of the variability. The components were labelled 'organizational support', 'team organization' and 'external change agent support'. One item not meeting item-scale criteria was removed. This resulted in a 14 item instrument. Scale reliability ranged from 0.77 to 0.91. Internal item consistency and divergent validity were satisfactory.

**Conclusion:**

On the whole, the instrument appears to be a promising tool for assessing team organization and internal and external support during QIC implementation. The psychometric properties were good and warrant application of the instrument for the evaluation of the national programme and similar improvement programmes.

## Background

In the past fifteen years enormous progress has been made in monitoring quality of care in the United States and several European countries. Monitoring may serve several purposes. It is often considered a prerequisite for organizational learning and a driver for ongoing development. The Continuous Quality Improvement (CQI) techniques that were introduced into health care in the 1980s, for instance, fit within this line of reasoning, as does the 'Breakthrough Series', launched in 1995 by the Institute for Healthcare Improvement (IHI) [1,2]. Both CQI and Breakthrough offer a baseline for realizing changes, but where the first one emphasizes that most quality problems are a result of system failures, the second approach regards them as problems with individual practitioners. In the Breakthrough view, change processes depend greatly on the role of individual professionals within the complex system of their working environment. The core technology of the approach involves the identification of deficiencies in quality, repeated implementation of small-scale interventions and measuring of changes, followed by refinement and expansion of the interventions to improve care processes [2].

Breakthrough is an example of a quality improvement collaborative (QIC). It is a means to stimulate improvement and an intentional spread strategy. A QIC brings together groups of practitioners from different healthcare organizations to work in a structured way to improve one aspect of the quality of their service. It involves them in a series of meetings to learn about best practice in the area chosen, about quality methods and change ideas, and to share their experiences of making changes in their own local setting [3]. Given the popularity of collaboratives, Øvretveit *et al*. urged for more research into the different types of QICs and their effectiveness, as well as linking QIC-practices explicitly to organizational and change management theory. Indeed, further study of processes and outcomes of QICs is desirable. QICs are complex, time consuming interventions and hard evidence on their effectiveness is limited [4-6]. The current study is conducted to contribute to a theory driven understanding of the process and effects of QIC implementation. Our purpose is to develop and test a measuring instrument for three central elements of QIC-implementation: 1) the organization of teams who join a QIC, 2) the degree of support these teams get from their own organization, and 3) the support given by the external consultants or change agents facilitating the QIC and its meetings.

The study is part of an independent evaluation of a national improvement and dissemination programme for hospitals in the Netherlands. Objectives of the programme are to enhance patient safety and logistics in 24 hospitals. Three groups of eight hospitals receive programme support for two years. In the first year multidisciplinary teams implement projects that are to be disseminated throughout their hospitals in later years [7,8]. The programme is a combination of six types of QICs, each with their own topic, programme targets and specific interventions (table 1). Implementation of each project type is supported by an external change agency staffed by change experts and consultants.

**Table 1:** QIC-projects and programme targets

***Quality domain***	***QIC-project***	***Programme targets***
*Patient logistics*	working without waiting lists (WWW)	- Access time for clinical consultation is less than a week
	operating theatre (OT)	- Increasing the productivity of operation theatres by 30%
	process redesign (PRD)	- Decreasing the total duration of diagnostics and treatment by 40–90%
		- Reducing length of in-hospital stay by 30%
		
*Patient safety*	medication safety (MS)	- Decreasing the number of medication errors by 50%
	pressure wounds (PW)	- The percentage of pressure wounds is lower than 5%
	postoperative wound infections (POWI)	- Decreasing postoperative wound infections by 50%

Besides the scientific goal, this study serves a more practical purpose. Knowledge on team organization and supportive conditions is of considerable value for parties involved in QIC-efforts. Hospital managers, project teams, change agents and public stakeholders may benefit from gathering tangible information for real-time adjustments. Furthermore, anticipating on future events, it is important to guarantee the applicability of the instrument for evaluation purposes in other collaborative programmes. This requires measuring the instrument's basic psychometric properties, such as reliability and validity, by testing it in a representative sample of project leaders of the multidisciplinary hospital teams.

The measuring instrument in the current study is based on team organization and internal and external support. Before going deeper into the methods we will elaborate some more on the nature of the three dimensions.

### Three dimensions and their characteristics

*Team organization *affects the teams joining a QIC. Cohen and Bailey defined a team as 'a collection of individuals who are interdependent in their tasks, who share responsibility for outcomes, who see themselves and who are seen by others as an intact social entity embedded in one or more larger social systems (for example, business unit or corporation), and who manage their relationships across organizational boundaries' (p. 241).[9] There is a general consensus in the literature that a team consists of two or more individuals, who have specific roles, perform interdependent tasks, are adaptable, and share a common goal.[10] To increase the effectiveness of teams it is important to establish timely, open and accurate communication among team members.[11] The notion that QIC-teams are responsible and in charge of the progress of the project [3] is in line with literature suggesting that operational decision making during implementation processes should be devolved to teams.[12]

#### Internal support

Other imperatives for team success are strong organizational support and integration with the organization's key values.[13] Within QICs internal or organizational support has to do with leadership, support and active involvement by top management.[12,14,15] There should be regular contact between team and organization leaders, and the innovation must fit within the goals of the management.[15] Øvretveit *et al*. even state that the topic should be of strategic importance to the organization.[3] Besides the presence of necessary means and instruments [16] many of the internal support tasks are to be executed by the strategic management in particular. Executives have to communicate a vision, or at least key values, throughout the organization. [17,18] They must also stimulate the organization's and employee's willingness to change.[19] Tasks such as these fall within the priority setting areas as defined by Reeleeder *et al*. i.e. foster vision, create alignment, develop relationships, live values and establish process.[20]

#### External support

The involvement of external change agents, arranging group meetings for teams of different organizations, is a typical QIC feature. Team training is a success factor for team based implementation.[13] Team training can be more effective than individual training especially when the learning is about a complex technology.[21] The purpose of a QIC is that teams are empowered and motivated to implement new working methods in order to alter a quality aspect of their care delivery. External change agents should provide teams with an applicable model together with high performance expectations.[22] This implies and requires a gap between a perceived and an actual situation, as well as outlining the potential added value of the innovation to the teams.[3] A second prerequisite is that teams joining the QIC have to gain information and skills that are new to them, otherwise an important argument for joining the QIC is void. The external support dimension is connected to the other two dimensions. The central topics of the collaboratives organized by the external change agents can be seen as the innovations that will determine team focus during the implementation process. The nature of these innovations should be congruent with the organizational key values as mentioned before. Although highly simplified, this is the mechanism by which new working methods are brought into the home organizations of the QIC-teams via the external change agents.

## Methods

### Instrument development

The study goal is to design an efficient instrument to gather information on the three dimensions. The instrument is designed to be filled out by the project leader of the multidisciplinary team joining the QIC in the middle or at the end of the project. The project leader is most likely to be confronted with internal and external support aspects. Furthermore, the project leader is acquainted with the functioning of the multidisciplinary team running the project.

Item content is based on the three dimensions and their characteristics. To enhance content validity, nine experts in human resource management, organizational psychology, patient safety, logistics and operations management, social medicine and health care management reviewed the first draft of the instrument. They were asked to judge the questions for appropriateness, clarity, completeness, question sequence, completion time and overall appearance. Questions with potential overlap in construct, others that were vague, ambiguous and redundant and some, which appeared irrelevant to the objectives of the study, were removed, resulting in a 15 item instrument. Questions are displayed in table 2, divided into team organization (TO), external change agency (EX), hospital organization (HO).

**Table 2:** Item descriptive statistics

**Item**	Description*	**Valid (%)**	**Mean (SD)**	**Median**	**Distribution of valid responses (%)**						
	*Team organization (TO)*				**1**	**2**	**3**	**4**	**5**	**6**	**7**
1	there is good communication and coordination	100.0	5.55 (1.09)	6.0	0.0	1.8	3.0	10.9	22.4	46.7	15.2
2	the division of tasks is perfectly clear	100.0	5.31 (1.07)	5.0	0.0	0.6	5.5	13.9	34.5	33.3	12.1
3	everyone is doing what he or she should do	99.4	5.05 (1.35)	5.0	1.2	1.2	14.0	14.0	26.2	30.9	12.2
4	is responsible for progress of project	99.4	5.30 (1.23)	6.0	0.6	1.8	7.3	12.8	23.8	41.5	12.2
5	is in charge of project implementation	100.0	5.37 (1.23)	6.0	0.6	1.8	7.9	7.9	27.9	39.4	14.5
	*External change agent support (EX)*										
6	is properly trained	99.4	4.32 (1.38)	4.0	1.2	9.8	16.5	28.0	21.3	19.5	3.7
7	at collaborative meetings I always gain valuable insights	99.4	4.24 (1.48)	4.0	2.4	11.6	20.1	17.1	26.2	18.9	3.7
8	external change agents provide sufficient support and instruments	99.4	4.52 (1.27)	5.0	0.6	6.7	12.8	26.8	29.3	20.1	3.7
9	external change agents raised high expectations about performance and improvement potential	98.8	4.83 (1.25)	5.0	0.6	3.7	10.4	22.7	27.6	30.1	4.9
10	external change agents made clear from the beginning what the goal of the project is and the best way to achieve it	99.4	4.76 (1.24)	5.0	1.2	3.7	9.8	23.8	31.1	26.2	4.3
	*Support from hospital organization (HO)*										
11	we see that the project is important to the strategic management	97.6	5.08 (1.49)	5.0	0.0	6.8	11.2	13	24.2	25.5	19.3
12	we see that the strategic management supports the project actively	98.2	4.75 (1.60)	5.0	4.3	6.2	9.3	21.0	22.8	22.8	13.6
13	the hospital gives the support we need in the department(s) to make the project a success	99.4	4.36 (1.49)	5.0	5.5	7.3	11.6	25.6	25.0	21.3	3.7
14	does everything in its power to increase the willingness to change	99.4	4.18 (1.55)	5.0	5.5	10.4	16.5	23.2	22.0	18.3	4.3
15	the board pays attention to the activities of the project team	95.8	4.66 (1.67)	5.0	4.4	9.5	10.8	17.7	15.8	31.6	10.1

* 1–6: '(In) the project team...' 11–12: 'In the department(s) where the project is implemented...'

Items were designed based on a 7-point Likert scale in which 1 corresponds to 'strongly disagree', 2 to 'disagree', 3 to 'slightly disagree', 4 to 'neutral', 5 to 'slightly agree', 6 to 'agree' and 7 to 'strongly agree'. The choice for a 7-point scale is based on the notion that this scale offers maximum information, discriminates well between respondent perceptions and is easy to interpret.

After the draft version was tested by five researchers it showed that the instrument was simple and straightforward to complete and not time consuming (approximately 10 minutes). The instrument also contains a standard set of socio demographic and job related questions addressing age, sex, education, position, date of birth. Extra background information was added in the form of two questions, addressing the number of team members and meetings since the start of the project.

### Sample and data collection

To investigate the suitability of the instrument in a QIC in Dutch hospital care, all the project leaders of the teams participating in the improvement programme were included as participants in the study. The testing fields were 24 hospitals spread all over the country. The central change agency granted permission to approach the project leaders in the programme hospitals. The project leaders of one hospital decided not to participate in the study because of the expected time burden.

All questionnaires were assigned a unique code to distinguish between organizations, project types and respondents. In the first group, comprised of eight hospitals, the study was conducted between July and September 2005, 77 questionnaires were sent by mail to the project leaders. In the second group, consisting of seven hospitals, the study was conducted between December 2006 and February 2007 and this time 71 questionnaires were sent by mail to the project leaders. The third group filled out the questionnaire between September 2007 and December 2007 89 questionnaires. The overall response rate was 71%, (168 out of 237 questionnaires). Instructions were provided via an accompanying letter describing the purpose of the study and stating that the participation was anonymous.

### Analyses

The sample was analyzed as a whole. Descriptive statistics and the response distribution for each item were calculated, in order to examine central tendency, variability and symmetry. Reliability and validity were investigated as main psychometric properties. Reliability i.e. how well items reflecting the same construct yield similar results, was tested via Cronbach's alpha coefficient. This is the most frequently used estimate of internal consistency. The higher the score, the more reliable the generated scale is. A minimum score of 0.70 is preferred.[23]

Content validity was addressed in the development stage to enlarge confidence that the instrument measures the aspects it was designed for. To support construct validity, principal component analysis was used to determine the underlying constructs, which explain significant portions of the variance. The factor loadings, i.e. the correlation coefficients between the items and the factors, were examined in order to explain the meaning of each construct. Tests of scaling assumptions, according to the multi trait/multi-item matrix [37], were used to confirm the structure found. This approach extends the logic of the multi-trait-multi-method technique [24] from the level of traits to the level of items.[25] To test item-internal consistency items were correlated with their scale corrected for overlap (a correlation corrected for overlap is the correlation of an item with the sum of the other items in the same scale; the bias of correlating an item with itself is thus removed). High item convergent validity was indicated if the item correlated considerably with the relevant scale. A threshold of 0.40 was used, proposed by Karlsson *et al*.[25] Low item divergent validity was indicated if an item correlated higher with any other scale than with the own scale. A matrix was computed with item-scale correlations and correlations were thereafter compared across scales. The criterion for significant difference was two standard errors.[26] All analyses were performed using SPSS 14.0.

## Results

Nearly half of the respondents (48%) consisted of medical professionals (mostly physicians, nursing staff and paramedics). Two other dominant function groups in the sample are: managers, department heads or team leaders (29%) and a third group of advisors, policy makers and administrative and quality personnel (23%). The majority was female (60%) and the mean age was 43 years. The projects are divided into pressure ulcers 17% (n = 28), medication safety 23% (n = 38), postoperative wound infections 7.9% (n = 16), operation theatre productivity 7.9% (n = 16), process redesign 21.2% (n = 35) and waiting lists 19.4% (n = 32). Response information is provided in table 2, which displays the items descriptive statistics.

**Table 3:** Rotated component matrices: 15 items

**Item**	**Description**	**Pattern matrix**			**Structure matrix**		
		**1**	**2**	**3**	**1**	**2**	**3**
12	strategic management supports project actively	**0.935**	-0.086	-0.022	**0.898**	0.240	0.230
14	does everything to increase willingness to change	**0.911**	-0.050	0.023	**0.900**	0.282	0.279
11	project is important to strategic management	**0.843**	-0.081	0.092	**0.841**	0.248	0.318
13	hospital gives the support needed in department(s) to make project successful	**0.834**	0.058	-0.019	**0.849**	0.350	0.247
15	pays attention to team activities	**0.732**	0.140	-0.092	**0.754**	0.372	0.169
6	proper team training	**0.466**	0.276	0.103	**0.595**	0.474	0.328
4	responsible for progress	0.023	**0.808**	-0.073	0.290	**0.794**	0.185
2	clear division of tasks	-0.036	**0.786**	0.166	0.294	**0.825**	0.399
1	good communication and coordination	-0.115	**0.779**	0.101	0.193	**0.769**	0.308
3	everyone is doing what he or she should do	0.091	**0.776**	-0.050	0.353	**0.793**	0.218
5	in charge of implementation	0.061	**0.768**	-0.155	0.288	**0.741**	0.101
8	sufficient support and instruments external change agents	-0.101	0.048	**0.845**	0.168	0.274	**0.830**
7	gain valuable insights at collaborative meetings	-0.028	0.001	**0.799**	0.210	0.239	**0.791**
10	external change agents made goal and clarified way to achieve it	0.035	0.123	**0.715**	0.292	0.357	**0.763**
9	external change agents raised high expectations about performance and improvement potential	0.151	-0.204	**0.708**	0.289	0.069	**0.690**

*Notes: *Extraction method: Principal Component Analysis. Rotation method: Promax with Kaiser normalization. Rotation converged in five iterations.

Valid responses are high for all items, providing evidence that items and response choices are clear and unambiguous. Three respondents had filled in less than half of the total number of items and were excluded from further analyses. When a respondent had given more than one option, this item was marked "missing". There were no items with 80% of the answers falling in one category. No items were excluded based on the percentage of missing responses.

The suitability of the data for component analysis was tested via the Kaiser-Meyer-Olkin measure of sampling adequacy, which tests the partial correlations among the items. Its value should be higher than 0.5 for a satisfactory analysis to proceed [27]. The KMO measure in this study was 0.82. Next, Bartlett's test of sphericity verified that the inter-item correlations were sufficient (X^2 ^= 1211.8; df = 105; P < 0.001). The correlation matrix is thus not an identity matrix, which would indicate that the factor model is inappropriate because variables only correlate with themselves and all other correlation coefficients are close to zero.[28] Principal Component Analysis (PCA) was chosen as the approach to establish which linear components exist within the data and how particular variables contribute to that component.

**Figure 1:**
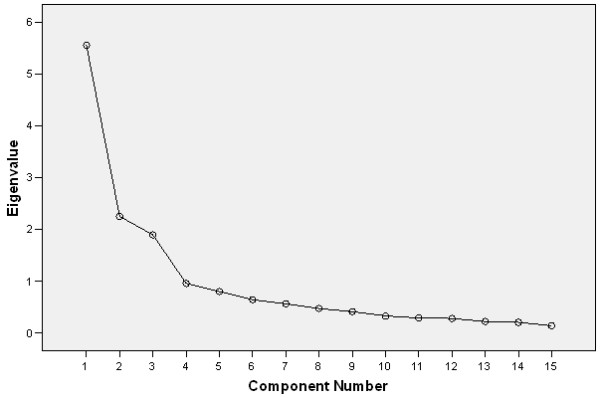
Cattell's scree plot; a two dimensional graph with factors on the x-axis and eigenvalues on the y-axis.

A decision to be made is the number of linear components or factors. A typical approach is the Kaiser-Guttman rule which states that an eigenvalue (i.e. the variance accounted for by each underlying factor) must be greater than one. However this approach usually produces many factors along with the inherent difficulty of properly interpreting them. Another eigenvalue-based approach is to examine Cattell's scree plot; a two dimensional graph with factors on the x-axis and eigenvalues on the y-axis. Based on the scree plot in figure 1 and the Kaiser-Guttman rule three factors can be identified.

Rotation maximizes the loading of each variable on one of the extracted components whilst minimizing the loading on all other components. The exact choice of rotation depends on the answer to the question whether or not underlying factors are related. When on theoretical grounds the components should be independent an orthogonal rotation like varimax is recommended. However, if theory suggests that factors might be correlated, then an oblique rotation is to be selected. DeVellis provides specific guidance for when an orthogonal rotation should be used. He suggests that when the correlations among the factors are less than .15, the orthogonal approach is best, but otherwise the oblique rotation is a better option.[29] Since we assume that the three dimensions in our study are related to each other, we prefer an oblique promax rotation over an orthogonal rotation. PCA demonstrated three factors cumulatively accounting for 65% of variation in all components. The first accounts for 37% of the variance, the second for 15% and the third for 13%.

Oblique rotation generates a pattern matrix with factor loadings and a structure matrix with correlations between items and components in a structure matrix. Table 3 contains both matrices. The structure matrix differs from the pattern matrix in the sense that shared variance between components is not ignored. The pattern matrix contains standardized regression coefficients (weights) which reflect the relative and independent contribution of each factor to the variance of the item on which it loads.[30] The structure matrix loading is a measure of the association (Pearson's correlation coefficient) between each item and the factor on which it loads, when the factors are correlated there is overlap among the loadings, which make structure matrix loadings biased estimates of the independent relationship between item and factor.[30] It is for this reason that our interpretation of the factors is based on the pattern matrix coefficients rather than the structure matrix loadings.

A cut-off point of 0.50 for factor loadings was adopted, i.e. only those items scoring higher than this threshold were retained for further analyses [31]. Item 6 "*team is properly trained*" was dropped based on this criterion. It was not necessary to apply a second criterion; none of the remaining items loaded higher than 0.4 on more than one factor.[28]

In table 4 the pattern and the structure matrix following from the component analysis are presented again, this time without item 6 and values < .40. The three components are labelled 'organizational support', 'team organization' and 'external change agent support'. For each component the reliability is assessed using Cronbach's alpha. Coefficients range from 0.77 to 0.91, higher than the preferred 0.70 level. In the right column the alpha value is shown for each component per item if that item would be deleted. Removing items does not lead to an improvement of the scale reliability of the components. In table 2 item 6 was distributed into external change agent support. Cronbach's alpha of the third component incorporating item 6 is 0.74. Adding item 6 does not improve scale reliability.

**Table 4:** Rotated component matrices and Cronbach's alpha: 14 items

**Item**	**Description**	**Pattern matrix**			**Structure matrix**			
		**1**	**2**	**3**	**1**	**2**	**3**	**Scale if item removed**
	**1^st ^FACTOR: organizational support (5 items; alpha = 0.91)**							
12	strategic management supports project actively	0.932			0.910			0.87
14	does everything to increase willingness to change	0.897			0.897			0.87
11	project is important to strategic management	0.840			0.850			0.89
13	hospital gives the support needed in department(s) to make project successful	0.821			0.844			0.89
15	pays attention to team activities	0.726			0.755			0.91
	**2^nd ^FACTOR: team organization (5 items; alpha = 0.84)**							
4	responsible for progress		0.811			0.805		0.81
2	clear division of tasks		0.780			0.819		0.80
3	everyone is doing what he or she should do		0.776			0.795		0.81
1	good communication and coordination		0.773			0.769		0.81
5	in charge of implementation		0.767			0.747		0.82
	**3^d ^FACTOR: external change agent support (4 items; alpha = 0.77)**							
8	sufficient support and instruments external change agents			0.842			0.830	0.67
7	gain valuable insights at collaborative meetings			0.797			0.791	0.71
10	external change agents made goal and clarified way to achieve it			0.714			0.762	0.72
9	external change agents raised high expectations about performance and improvement potential			0.704			0.694	0.76

*Notes: *Extraction method: Principal Component Analysis. Rotation method: Promax with Kaiser normalization. Rotation converged in five iterations.

The 14 items were used to create the multi trait/multi-item matrix shown in table 5. The matrix helps to examine the relationship of each item with its own scale, as well as its correlations with other scales. Item-scale convergent validity is tested by checking the range of item-scale correlations. Item-internal consistency is satisfactory and the inclusive criterion of a correlation of 0.40 or higher is met for all items. The multi trait/multi-item correlation matrix also allows examination of the assumption that items are stronger measures of their constructs than of the other constructs. In order to be significant the item-scale correlation for a scale should be at least two standard errors higher. The standard error of the correlation coefficient is approximately equal to 1 divided by the square root of the sample size. In our case two standard errors is equal to: 2(1/√165) = 0.16. For all three factors the divergent validity test demonstrated significant success.

**Table 5:** Summary of results of multi-trait/multi-item scaling

	**Item-scale convergent validity Criterion 1 (inclusive criterion)**		**Item-scale divergent validity Criterion 2 (exclusive criterion)**		**Scaling fulfilment**
**Scale**	Range of item-scale correlations^1^	Number of item-scale correlations^2^	Range of correlations with other scales^3^	Number of items higher correlation with other scale^4^	Number of items that meet criterion 1 but not 2
1. Organizational support	0.646–0.833	5/5	0.165–0.371	0/5	0/5
2. Team organization	0.601–0.701	5/5	0.096–0.392	0/5	0/5
3. External change agent support	0.471–0.657	4/4	0.070–0.350	0/4	0/5

1 Pearson correlations between items and hypothesized scale (corrected for overlap).

2 Number of item-scale correlations that meet minimum standard for convergent validity (≤ 0.40).

3 Pearson correlations between items and other scales.

4 Correlations higher between items and other scales in comparison with hypothesized scale (by two standard errors or more; ≤ 0.16)

## Discussion

Before going deeper into the interpretation and implication of the components found, the steps taken so far will be summarized. The theoretical framework of this study is built on literature about QICs, team based implementation and the dissemination of innovations within health service organizations. Appropriateness, clarity and completeness of the items in a draft version of the instrument was revised by experts who also judged the appearance, question sequence and completion time. This step was an important exercise for supporting content validity and resulted in a 15 item questionnaire that was administered by project leaders of a national hospital care improvement programme, 165 of the returned questionnaires (70%) were included in the study. Principal component analysis was performed and three components were extracted, accounting for 65% of the variance of the items. Item-scale criterion was not satisfied in the case of one item, which was eventually excluded from the instrument. Construct validity was supported by the overall success of the convergent and discriminant validity tests of item-scale correlations, according to the multi trait/multi-item correlation matrix approach. Reliability of the three components was addressed using Cronbach's alpha coefficient, which was well above the recommended minimum value for each individual construct.

The first factor contains five organizational support items. The second factor also consists of five items, now affecting the organization of the project team. The four items of the third factor relate to the support given by external change agents. The factor structure found in the data is almost identical to the three subcategories in table 2 (left column). However, instead of what we expected 'proper team training' loaded on organizational and not on external change agent support.

A few remarks must be made with regards to the sample size. In the literature different standards are applied for the number of items-number of cases ratio for a factor or principal component analysis. Kass and Tinsley recommend five to ten cases for each item.[32] Nunnally is more restrictive and recommends at least ten, a threshold met in this study.[33] A second point is the total sample size. There is no real consensus in the literature on this criterion. Several authors consider 300 cases as comforting [34,35], 100 as poor and 1000 as excellent [35]. Nevertheless, according to MacCallum *et al*. samples between 100 and 200 can be good enough provided that communalities are higher than .5 and there are relatively few factors each with only a small number of indicator variables.[36] The lowest communality in this study was .52 with a minimum factor size of four. Guadagnoli and Velicer state that the most important issues in determining reliable factor solutions are the absolute sample size and the absolute magnitude of factor loadings. In short, they argue that if a factor has four or more loadings greater than .6 then it is reliable regardless of sample size.[37]

A limitation of this study is that we could not assess Test-Retest Reliability due to an agreement made between the funding organization, hospitals, programme makers that the questionnaire burden for hospital staff was to be minimized. Another possible limitation is that the instrument was tested for its overall psychometric properties using the combined sample of project leaders. The respondents differ in position, type of project, hospital organization and time period – the first year of the first group, took place a year before the first year of the second group and two year before the first year of the third group. Despite these differences, it is likely that the instrument is suitable for the evaluation of other collaborative health care improvement programmes. The instrument was tested for its overall psychometric properties using the combined sample of project leaders. Notwithstanding functional and other differences the study results show that the respondents could very well make a distinction between the three dimensions. The three components form a basic measuring instrument and a promising step towards a better understanding of QIC-implementation. Combined with the qualitative methods that are indispensable for a programme evaluation, the quantitative data gathered using the instrument can potentially add more detailed information on the relations between the components and narrative data collected by interviews or observations.

This study reported on the development and psychometric testing of a measuring instrument. A short term benefit from measuring the conditions during the implementation is that it may be helpful in identifying those project teams with deficiencies in the areas measured by the instrument, in order to provide them with additional resources and support. Yet, more fundamental questions may be answered using data from this questionnaire. Insight into team organization and support during the implementation may help in understanding how process features affect the actual or perceived amount of success. There is, nonetheless, something to gain by adding supplementary questions, i.e. on the complexity, relative advantage and the nature of the specific interventions that are implemented by the teams, as well as the indicators used to monitor the project targets. Other relevant questions affect scales measuring the learning climate within the implementation area, activities taken on behalf of sustaining new working methods, the quantitative spread of the projects throughout the organization and so on. Extensions like these can be rewarding in an a priori fashion, since they potentially illuminate the complexity and advantage brought by a project in relation to the types of interventions and the measuring efforts that are part of applying rapid cycle improvement. At this moment knowledge on these matters is limited but very welcome.

## Conclusion

This study resulted in the development of a measuring instrument for team organization and supportive conditions for the implementation of QIC projects. After psychometric testing it demonstrated acceptable levels of internal consistency reliability and content and construct validity. This evidence warrants application of the instrument for the evaluation in the hospital improvement programme and similar QICs in health care. Linking outcome data on performance indicators to the state of the conditions during the implementation may be helpful in explaining, perhaps even predicting, the amount of success.

## Competing interests

The authors declare that they have no competing interests.

## Authors' contributions

MLAD was responsible for designing the study, conducting the literature review, developing the questionnaire, acquiring, analyzing and interpreting the data and drafting the manuscript. As research manager of the independent evaluation study of the hospital improvement programme CW was responsible for designing the study and developing the questionnaire. CW and PPG assisted in interpreting the results and revising the manuscript for intellectual content. All authors have read and approved the final manuscript.

## Pre-publication history

The pre-publication history for this paper can be accessed here:


